# Prevalence of Cefepime-Resistant *Escherichia coli* in Iran: A Meta-Analysis (2007–2016)

**Published:** 2019-04

**Authors:** Seyyede Maryam BECHASHK, Ghobad MORADI, Behzad MOHSENPOUR, Rashid RAMAZANZADEH

**Affiliations:** 1. Student Research Committee, Kurdistan University of Medical Sciences, Sanandaj, Iran; 2. Social Determinants of Health Research Center, Research Institute for Health Development, Kurdistan University of Medical Sciences, Sanandaj, Iran; 3. Department of Infectious Diseases, Faculty of Medicine, Kurdistan University of Medical Sciences, Sanandaj, Iran; 4. Cellular & Molecular Research Center, Research Institute for Health Development, Kurdistan University of Medical Sciences, Sanandaj, Iran; 5. Department of Microbiology, Faculty of Medicine, Kurdistan University of Medical Sciences, Sanandaj, Iran

**Keywords:** Prevalence, Antimicrobial resistance, *Escherichia coli*, Cefepime

## Abstract

**Background:**

Cefepime-resistant *Escherichia coli (E. coli)* is one of the seven important types of microbial resistance. This meta-analysis study was conducted to identify the prevalence of cefepime-resistant *E. coli* in Iran during 2007–2016.

**Methods:**

Studies published were searched in Persian databases (including Magiran, and SID) and international databases (including PubMed, ScienceDirect, and Scopus). The selected studies included all types of laboratory methods. Moreover, the random effects model (DerSimonian Laird method) was used to addresses the high heterogeneity (50% < I^2^) between the reviewed studies. The collected data were categorized into different subgroups on the basis of the year of study and province. Data analysis was performed using the Statsdirect software.

**Results:**

Overall, 516 articles were selected from the searched medical databases. After reviewing and applying the inclusion criteria, irrelevant papers were excluded and the remaining 26 studies were meta-analyzed. The overall prevalence of cefepime-resistant *E. coli* was 53.42% (95%: 43.35, 63.35), ranging from 25% (95%CI: 21.67, 28.55) in 2009 to 61.95% (95%CI: 56.62, 67.09) in 2016.

**Conclusion:**

The prevalence of cefepime-resistant *E. coli* in Iran has had an increasing and alarming trend during the recent years. Therefore, it is necessary to use practical strategies and interventions to control and monitor cefepime-resistant *E. coli* in the country.

## Introduction

Antimicrobial resistance (AMR) is one of the most important challenges facing the public health and is introduced by (WHO) as a growing major health threat. Given the increasing spread of AMR in the world, the WHO recommends controlling and monitoring AMR and the use of antibiotics (1, 2). Cefepime-resistant *E. coli* is one of the most important germs. *Escherichia coli* is a gram negative bacteria, anaerobic, spore free, and normal flora that is the main cause of more than 80% of the acquired urinary tract infections (3). Infection caused by various strains of *E. coli* results in a wide range of digestive symptoms, including fever, headaches, and diarrhea in children and adults in developing and developed countries (4). Diarrheagenic *E. coli* can cause childhood diarrhea, traveler's diarrhea, dysentery, and hemolytic uremic syndrome. The infectious disease caused by cefepime-resistant *E. coli* is spreading worldwide and about 50% to 60% of nosocomial infections caused by *E. coli* are cefepime-resistant (5, 6).

The consumption of contaminated water and food, fecal contamination, and person-to-person transmission are the main routs of *E. coli* transmission (7). Resistance to the third generation of cephalosporin in six regions of WHO is about 50% to 80% (8).

Infections caused by *E. coli* lead to an increase in the costs of admission and the mortality from diarrhea in children and adults, especially in developing countries (9). The costs associated with hospitalization and treatment could be reduced through considering geographical and climatic differences, improving the surveillance system, and epidemiological monitoring of diseases (10).

Seven AMRs, including *E. coli*, *Klebsiella pneumonia*, Methicillin-resistant *Staphylococcus aureus*, *Shigella* species, *Neisseria gonorrhea*, *Clostridium difficile*, and *Pseudomonas* are the major threats and challenges to the public health (11–13).

This study aimed at evaluating the prevalence of cefepime-resistant *E. coli* in Iran through the meta-analysis of studies conducted from 2007 to 2016.

## Methods

### Search strategy

The present study was conducted on the available papers published in the Persian and international databases. Accordingly, using a selected group of keywords, the Persian databases including Magiran and SID and the English databases, including PubMed, ScienceDirect, and Scopus were searched comprehensively to find studies conducted on antimicrobial-resistant *E. coli* published from 2007 to 2016.

### Inclusion and exclusion criteria

Inclusion criteria: All cross-sectional studies in Persian and English on the prevalence of cefepime-resistant *E. coli* from 2007 to 2016 in Iran were included. Moreover, studies conducted on human samples and Iranian population were included.

Exclusion criteria: Studies that did not estimate the prevalence of cefepime-resistant *E. coli* and were not related to the topic and title of the study were excluded. Non-cross-sectional studies (review, meta-analysis, case-control, and cohort studies), studies performed on non-humane samples, and studies with duplicated data were also excluded.

### Selection of studies

Initially, the Persian and English databases were separately searched by two of the researchers. To select the related papers, their title, then their abstracts, and finally their full text were reviewed. At each stage, in case of controversy, consensus was achieved through consulting with the supervisor of the research team to make a final decision. The lists of references of the selected studies were also evaluated and the relevant articles were included in the study. All studies that estimated the prevalence of cefepime-resistant *E. coli* without especial antimicrobial susceptibility test type were included.

### Collected data

Data on authors’ name, date of publication, time of study (year), place and type of study, sample size, gender, mean age of samples, and type of antibiotic resistance were extracted from the selected cross-sectional studies performed on human samples. The collected data was entered into Statsdirect software for final analysis.

### Quality of studies

Strobe checklist was used to assess publication bias and quality of the selected studies (14). Strobe checklist has 22 items categorized into seven categories. Items used include the following: reference to the study design, expression of the method of exposure and outcome measurement, method of calculating the sample size, flowchart for selecting the subjects, reference to the time of data collection, reference to inclusion and exclusion criteria.

After evaluating the aforementioned cases, the studies were divided into three groups: studies with high, moderate, and low bias. Low-bias and high-quality studies are studies that meet all the seven items listed in Strobe checklist. Studies with moderate bias and moderate quality are studies that meet six items of Strobe checklist. Studies with high bias and low quality are studies that do not meet two or more items of Strobe checklist. The criteria used to measure bias in the selected studies include: bias in outcome evaluation, bias in exposure assessment, sample size calculation, information bias, and selection bias. All stages of the quality assessment of the articles were also conducted by two researchers independently. PRISMA checklist (2009) was used to assess the quality of systematic review and meta-analysis (15).

### Information analysis

This study aimed at evaluating the prevalence of cefepime-resistant *E. coli* calculated as *P* (ratio) with a confidence interval (CI) of 95%. To test heterogeneity, Q test was used at an error level of less than 10%, and its quantity was calculated using I^2^ index. I^2^ is an index used for estimating the variance between studies. Considering the results of heterogeneity test, random effects model was used for data analysis at a confidence interval of 95% (16). Given the high heterogeneity (50%<I^2^) in the study, the data was analyzed using DerSimonian Laird method. Data analysis was also performed on subgroups there were determined based on the province of residence and year of study. Beg rank correlation test was used to measure publication bias. A *P*-value<0.1 indicates a significant bias. A funnel plot was used to evaluate the publication bias. In addition, a significance level of α=0.05 was used in bilateral statistical tests (17, 18). After extracting the data from the articles using Statsdirect software, a meta-analysis was performed on the extracted data. Random effects model was used for subsequent studies

## Results

**Fig. 1:**
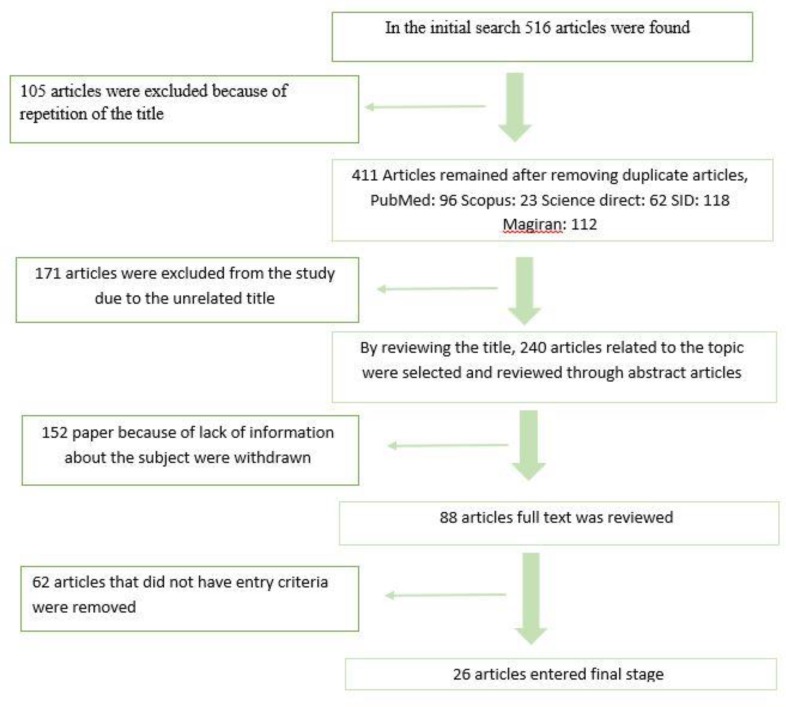
Flow of data through the different phases of the systematic review

Overall, 516 articles were found through searching the selected databases. Of all, 105 articles were excluded because they were duplicated. Of the 411 remaining articles, 171 whose titles were not related to the topic of the study were also excluded from our systematic review. After reviewing the titles and abstracts of the remaining articles, 240 ones related to the topic were selected for reviewing the full-text of the papers, of which 152 were excluded due to the lack of access to their full texts. Finally, the full texts of 88 articles were reviewed and 62 articles that did not meet the inclusion criteria were excluded from our study. At the end, the 26 remaining studies underwent the meta-analysis phase (Fig. 1).

### Study features

All these studies had been conducted in Iran and reported the prevalence of cefepime-resistant *E. coli* in the provinces of Tehran, Kurdistan, East Azerbaijan, Esfahan, Mazandaran, Kerman, Markazi, Guilan, and Ilam. The sample size varied from 13 to 504 cases (Table 1).

**Table 1:** Characteristics of studies on the prevalence of cefepime-resistant *E. coli*

***First Author***	***Publication Year***	***Province***	***Survey Year***	***Prevalence(%)(%95CI)***	***Sample(N)***
Zahra Babaei	2012	Tehran	2010	95.12(89.68-98.18)	123
Mozhgan Mohammadi	2011	Ilam	2007–2008	68(62.35–86.51)	54
Reza Dehbanipour	2016	Esfahan	2013–2014	50(20.08–35.74)	135
Hayedeh Mobin	2009	Azarbayjan Sharghi	2008	70(49.99–83.88)	32
Reza Mohebbi	2009	Ilam	2007	16(9–24.67)	100
Hossein Kaviani	2012	Tehran	2010–2011	44.82(26.44–64.30)	29
Toloe Bahhaei	2014	Guilan	2012	62(42.13–77.09)	33
Majid Eslami	2012	Tehran	2011	36(29.35–43.07)	200
Yosef Ramezani	2015	Tehran	2014	70.90(57.10–82.37)	55
Nahid Soleimani fard	2014	Arak	2013	70(55.39–82.13)	50
Seyyede sara Mosavi	2015	Kurdistan	2014	32(51.74–71.52)	100
Kolsome Asadpour	2015	Guilan	2013–2014	31.79(25.32–38.82)	195
Mahdi Mobasheri Zadeh	2015	Esfahan	2013	63.33(54.05–71.94)	120
Majid Parnori	2010	Azarbayjan Sharghi	2008	65.85(49.40–79.91)	41
Alireza Mobasherkar	2008	Azarbayjan Sharghi	2007	65.85(49.40–79.91)	41
Hamid Salaki	2016	Tehran	2012–2013	38(48.95–76.37)	52
Elahe Ferdosi	2015	Mazandaran	2013	22.80(12.73–35.83)	57
Mohammad sadegh Rezaei	2015	Mazandaran	2013	66.97(61.58–72.04)	327
Ahmad Alikhani	2015	Mazandaran	2013	100(85.75–100)	24
Narges Najafi	2013	Mazandaran	2009–2011	100(75.29–100)	13
F Khorosh	2008	Esfahan	2005–2006	24(8–42.25)	27
Abolfazl gholipour	2014	Esfahan	2011–2012	31.83(26.05–38.06)	245
N Adib	2014	Kerman	2009	15.32(9–22.47)	137
Azar Haddadi	2008	Tehran	2004–2005	68(46.49–85.05)	25
Maryam Hafifpanah	2016	Guilan	2014	65(57.07–72.36)	160
Safar Farajnia	2009	Azarbayjan Sharghi	2008	24(20.34–27.98)	504
Total				53.43(43.35–63.35)	2879

### Prevalence

**Fig. 2:**
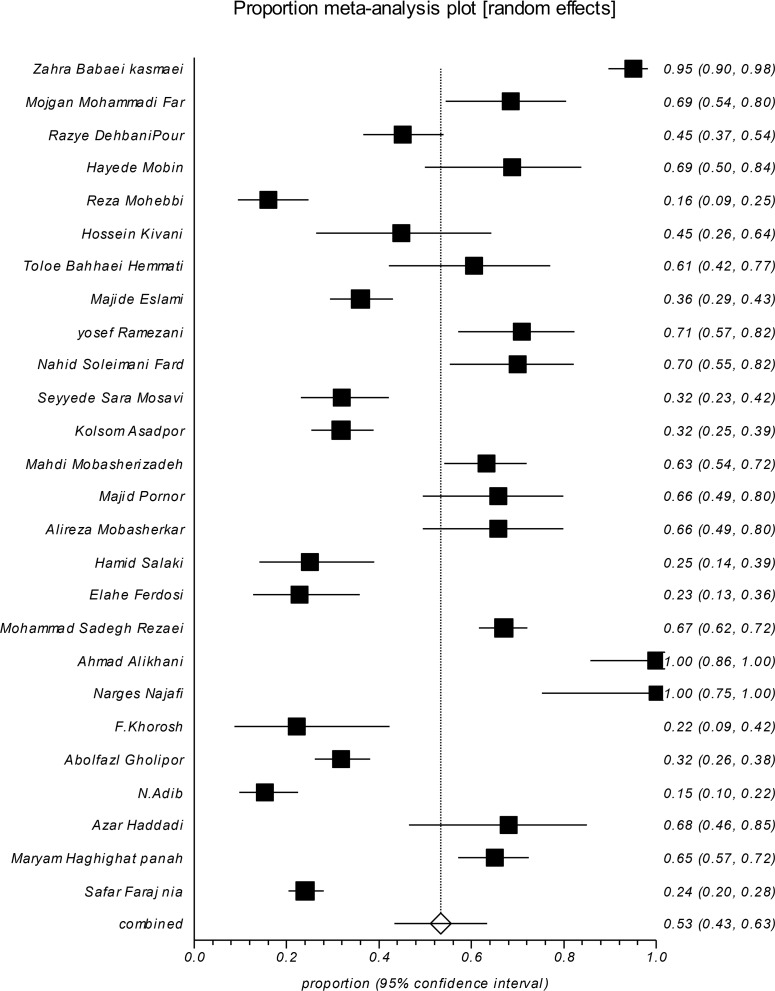
Forest plot of the prevalence of cefepime-resistant *E. coli* reported in different studies in Iran from 2007 to 2016

Twenty six studies reported the prevalence of cefepime-resistant *E. coli* in Iran from 2007 to 2016. Overall prevalence of cefepime-resistant *E. coli* was 53.42% (95% CI: 43.35, 63.35). The level of heterogeneity in the prevalence rates was 96.5%, which is consistent with the Cochrane definition in 2008 (19) (Fig. 2).

### Subgroup analysis

Considering year of publication, the prevalence of cefepime-resistant *E. coli* was 61.95% (56.62, 67.09) in 2016 and 25 % (95%CI: 21.67, 28.55) in 2009. The heterogeneity among the studies (*P*<0.001, I^2^=96.6%) was calculated through the quantitative evaluation via Beg test= (z=0.1, *P-*value=0.476). Considering province of study, the prevalence of cefepime-resistant *E. coli* was estimated to be 70% (95%CI: 55.39, 82.13) in Markazi and 15.32% (95%CI: 9, 22.47) in Kerman and there was a heterogeneity between the studies (*P*<0.001, I^2^=96.8%) (Table 2).

**Table 2:** Prevalence of cefepime-resistant *E. coli* By Subgroups

***Stratified factors***	***Prevalence (%)***	***Lower limit (%)***	***Upperlimit (%)***	***Model***
**Province**				
Tehran	60.12	55.60	64.51	Random
Ilam	37.01	29.38	45.15	Random
Esfahan	37.38	33.23	41.66	Random
Azarbayjane Sharghi	31.87	28.21	35.71	Random
Guilan	47.93	42.87	53.03	Random
Markazi	70	55.39	82.13	Random
Kurdistan	62	51.74	71.52	Random
Mazandaran	63.89	59.10	68.49	Random
Kerman	15.32	9	22.47	Random
**Year**				
2016	61.95	56.62	67.09	Random
2015	52.96	49.59	56.30	Random
2014	33.11	28.85	37.60	Random
2013	53.84	25.13	80.77	Random
2012	57.38	52.03	62.61	Random
2011	61.11	46.87	74.08	Random
2010	58.38	42.10	73.68	Random
2009	25	21.67	28.55	Random
2008	53.76	43.11	64.16	Random

### Publication bias

A funnel graph was used to show the distribution bias in studies conducted on the prevalence of cefepime-resistant *E. coli*. Beg test was used for quantitative evaluation of the publication bias (z=0.09, *P*-value=0.494).

### Sensitivity analysis

**Fig. 3:**
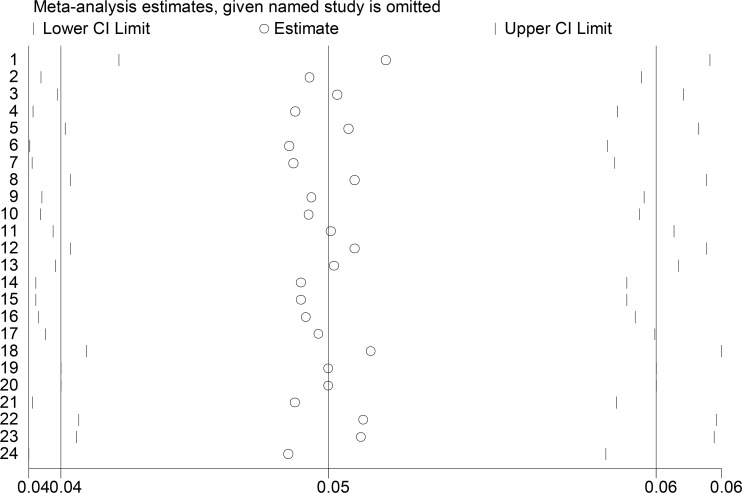
Sensitivity analysis of the prevalence of cefepime-resistant *E. coli*. (A) Results were computed by omitting each study one by one (B) The two ends of the dotted lines represent the 95% CI

To evaluate the impact of each study on the overall prevalence of cefepime-resistant *E. coli* at a confidence interval of 95%, each study was excluded and the results were compared with and without the results obtained through the analysis of all the studies. After the exclusion of each of the studies one by one, the results of susceptibility analysis showed that none of the studies alone had a significant effect on the overall prevalence (Fig. 3).

## Discussion

Twenty six studies had been conducted to determine the prevalence of cefepime-resistant *E. coli* in different provinces of Iran from 2007 to 2016. The sample size varied from 13 to 504 cases (20, 21). The overall prevalence of cefepime-resistant *E. coli* was 53.42% (95% CI: 43.35, 63.35). The lowest and highest sample sizes were observed in the provinces of Mazandaran and East Azerbaijan, respectively. From among the ten studied years, the highest prevalence rate was observed in 2016, indicating an increase in the resistance to antibiotics in recent decades. The highest and lowest prevalence rates, respectively, were 61.95% (95%CI: 56.62, 67.07) in 2016 and 25% (95%CI: 21.67, 28.55) in 2007.

The prevalence of cefepime-resistant *E. coli* in Iran was inconstant and varied from 15.32% to 100% (20, 22, 23). The prevalence of cefepime-resistant *E. coli* is different in various parts of the world. Its prevalence is estimated to be 10.3% in America, 8.8% in Europe, 6% in Argentina, and 13% in India (24–27). Among the countries located in the Southeast Asian region, the prevalence of cefepime-resistant *E. coli* is 0 in Taiwan and 13.5% in China (28, 29). As compared with neighboring countries in the Eastern Mediterranean region, Iran has a higher prevalence rate. The prevalence of cefepime-resistant *E. coli* is reported to be 12% in Saudi Arabia, 0 in Egypt, and 13% in Turkey (30–32). This difference in the prevalence could be attributed to the misuse of drugs, absence of some new antibiotics in the treatment protocol of some countries, and lack of appropriate monitoring and evaluation systems.

The prevalence of cefepime-resistant *E. coli* was 1% in Brazil (33). In the African regions, the prevalence of cefepime resistant *E. coli* was 8.3% in Ghana and 64.5% in Bamako (34). The prevalence of cefepime resistant *E. coli* in Iran is higher than that in countries located in European, American, and Eastern Mediterranean regions. The prevalence of resistance to this bacterium in Iran is high, more than the mean rate observed in the world and most countries. In addition to the aforementioned items, excessive consumption of antibiotics, lack of a proper surveillance system to monitor AMR, and lack of proper protocols to reduce and control antimicrobial resistance in Iran contribute to the high prevalence rate in Iran. Comparing the results of our study with those of other studies indicates a high prevalence of cefepime resistant *E. coli* in the country. The observed difference and the increased prevalence of cefepime resistant *E. coli* in various regions of Iran can be attributed to differences in diagnostic and laboratory conditions, indiscriminate use of drugs, and interrupted courses of drug use by patients. Undoubtedly, before the spread of the resistant types of the microbe throughout the country, they become more prevalent in some places, and they gradually spread to other parts of the country. It is necessary to prevent the increase in the prevalence of cefepime resistant *E. coli* in the country through training people, raising public awareness, monitoring the process of prescribing antibiotics and correct use of them. Today, the phenomenon of antimicrobial resistance is one of the major concerns of the WHO. According to the results of this study, the highest prevalence was reported in a study performed in Mazandaran with a sample size of 24 cases, in which the prevalence was estimated to be 100% (95%CI: 85.87, 100) (23). In Mazandaran with a sample size of 13 cases, the prevalence of was estimated to be 100% (95%CI: 75.29, 100) (20). The lowest prevalence was observed in a study in Kerman with a sample size of 137 cases, in which the prevalence of cefepime-resistant *E. coli* was 15.32% (95%CI: 9, 22.47) (22). The overall prevalence rate in Iran was estimated to be about 53% (95%CI: 43.35, 63.35). The prevalence rates varied in different provinces. Special attention should be paid to areas with a higher prevalence. Methods of antibiotics prescription in different parts of the country may differ to some extent. Despite the fact that the studied microbe is reported to be one of the seven priorities of the WHO, few studies have investigated it. Researchers are suggested to study the microbe using more precise methods to measure AMR.

This study had some limitations, for instance no specific species of *E. coli* was studied. In all provinces of Iran, no study had been performed on the prevalence of cefepime-resistant *E. coli.* However, an overall estimation in the country could be obtained from the prevalence rates reported in the nine studied provinces. In all the studied years, no study was performed on cefepime-resistant *E. coli*. In these studies, no special and unique laboratory method was applied. Moreover, in meta-analysis studies, there is always the possibility of losing some articles taken into account.

## Conclusion

The prevalence of cefepime-resistant *E. coli* in Iran is high and has an increasing trend. This increasing trend is a challenge to be resolved. It is necessary to adopt appropriate measures and interventions.

## Ethical considerations

Ethical issues (Including plagiarism, informed consent, misconduct, data fabrication and/or falsification, double publication and/or submission, redundancy, etc.) have been completely observed by the authors.
